# Framing Binational Challenges of Mental and Cognitive Health Care in Mexican-Origin Older Adults: Consensus Findings

**DOI:** 10.1093/geroni/igab046.1845

**Published:** 2021-12-17

**Authors:** Stephanie Grasso, Flavia Andrade, Mariana López-Ortega, Emma Aguila

**Affiliations:** 1 University of Texas at Austin, The University of Texas at Austin, Texas, United States; 2 University of Illinois at Urbana-Champaign, University Of Illinois At Urbana-Champaign, Illinois, United States; 3 National Institute of Geriatrics, National Institutes of Health, National Institute Of Geriatrics, National Institutes Of Health, Distrito Federal, Mexico; 4 Sol Price School of Public Policy, University of Southern California, California, United States

## Abstract

Growth in older populations, and hence in the number of persons living with dementia, is particularly rapid for individuals of Mexican origin living in the U.S. and Mexico. In order to identify influences on cognitive health in this diverse population, the University Texas at Austin and Mexican National Institute of Geriatrics (INGER) organized their second Bridging Conference titled: "Framing Challenges of Cognitive and Mental Health Care in Mexican-origin Older Adults in Mexico and the U.S". In this presentation, we highlight the results of a consensus-building session, during which bi-national expert opinions were generated and synthesized addressing gaps in research, knowledge, and policy, as well as the setting of priorities for immediate action and future research. Reducing barriers to adequate care for those aging-in-place with dementia was a central theme of the identified priorities. Critical areas of identified need, more specifically, included reducing social isolation, caregiver burden, and diminishing retirement income.

